# Screening of Differentially Expressed Genes and Localization Analysis of Female Gametophyte at the Free Nuclear Mitosis Stage in *Pinus tabuliformis* Carr.

**DOI:** 10.3390/ijms23031915

**Published:** 2022-02-08

**Authors:** Zaixin Gong, Hailin Hu, Li Xu, Yuanyuan Zhao, Caixia Zheng

**Affiliations:** 1 College of Biological Sciences and Technology, Beijing Forestry University, Beijing 100083, China; gongzaixin@jlau.edu.cn (Z.G.); huhailin@bjfu.edu.cn (H.H.); xuli1@bjfu.edu.cn (L.X.); 2 College of Horticulture, Jilin Agriculture University, Changchun 130118, China

**Keywords:** *Pinus tabuliformis*, female infertility, transcriptome sequencing, plant hormones, fluorescence in situ hybridization (FISH)

## Abstract

Female sterility is a common phenomenon in the plant world, and systematic research has not been carried out in gymnosperms. In this study, the ovules of No. 28 sterile line and No. 15 fertile line *Pinus tabuliformis* were used as materials, and a total of 18 cDNA libraries were sequenced by the HiSeqTM 4000 platform to analyze the differentially expressed genes (DEGs) and simple sequence repeats (SSRs) between the two lines. In addition, this study further analyzed the DEGs involved in the signal transduction of plant hormones, revealing that the signal pathways related to auxin, cytokinin, and gibberellin were blocked in the sterile ovule. Additionally, real-time fluorescent quantitative PCR verified that the expression trend of DEGs related to plant hormones was consistent with the results of high-throughput sequencing. Frozen sections and fluorescence in situ hybridization (FISH) were used to study the temporal and spatial expression patterns of *PtRab* in the ovules of *P. tabuliformis*. It was found that *PtRab* was significantly expressed in female gametophytes and rarely expressed in the surrounding diploid tissues. This study further explained the molecular regulation mechanism of female sterility in *P. tabuliformis*, preliminarily mining the key factors of ovule abortion in gymnosperms at the transcriptional level.

## 1. Introduction

Sexual reproduction is the most evolved reproductive mode of plants. The development of embryos and reproductive organs is the key step to completing sexual reproduction. However, in the plant kingdom, female sterility is widespread during sexual reproduction, which might be induced by incomplete megagametophyte development, abnormal ovule development, the wrong number of polar nuclei, meiosis mitotic disorder, and some other factors. For example, the meiosis of a megaspore mother cell was abnormal in soybean, which led to damaged megaspore formation and female sterility [1]. Chen et al. reported that ovule development was damaged after the dysplasia of the inner integument primordium in *Punica granatum* L. [2]. Awasthi et al. found that the number of polar nuclei in megagametophytes was reduced in rice, and the unusual cell cycle progression caused disordered mitotic division during the formation of ovules [3]. These results showed that the mechanism of female sterility in plants is complicated. Furthermore, revealing the molecular regulation mechanism of female sterility is of great significance for the improvement of germplasm resources and the breeding of plus trees.

In the past few decades, many genes related to plant hormones participating in the regulation of ovule development have been reported, such as the ectopic expression of *YUC1* damaging the synthesis of auxin and disrupting cell specificity in the female gametophyte (FG) [4,5]. The PIN-FORMED (PIN) auxin protein family is the main efflux carrier, and in *Arabidopsis*, low expression of *PIN1* might lead to mitosis arrest and female sterility [6]. Cytokinin oxidases/dehydrogenases (CKX) are related to hormone concentrations and the degradation of cytokinin, whose overexpression would reduce ovule abortion in *Arabidopsis* [7,8]. Most of the studies about plant sterility are focused on plants with a short life cycle [9], while studies of woody plants, especially gymnosperms, are limited.

Gymnosperms combine some features of spore plants and angiosperms, which plays an important role in the plant evolutionarily, thus benefiting the study of their reproductive development [10]. Conifers dominate the world’s forest ecosystems and are the most widely planted tree species, accounting for more than 70% of timber in the world [11]. *Pinus tabuliformis* Carr. is a unique evergreen conifer species in China and has important ecological and garden ornamental value [12]. In addition, *P. tabuliformis* takes 13 months to complete the process from the beginning of free nuclear mitosis of megagametophyte (FNMM) to complete cellularization; therefore, *P. tabuliformis* is a suitable material for the study of molecular regulation in gymnosperm reproductive development [13]. Our previous research found that line No. 28 was a female sterile mutated line in the *P. tabuliformis* seed orchard in Xingcheng, Liaoning, China, of which the appearance of the cone was normal, but FNMM was terminated and resulted in ovule abortion [14]. To reveal the reason behind female sterility in *P. tabuliformis*, we have carried out a series of systematic studies based on anatomy [15], physiology and biochemistry [16], and proteome level [17] and obtained a series of meaningful results. However, because the factors of ovule abortion were polyphyletic and the available techniques were limited, the regulatory mechanisms of ovule development are still unclear and need to be further researched.

In the present study, we used high-throughput sequencing and bioinformatics technologies to analyze the gene expression levels in different samples to reveal the mechanism of *P. tabuliformis* female sterility during the process of free nuclear mitosis (FNM). Furthermore, we also screened out potential differentially expressed genes (DEGs) participating in the regulation of ovule abortion during the FNM process by cluster analysis. In addition, the distribution characteristics and composition of SSRs in the ovules were also counted. Fluorescence in situ hybridization (FISH) was used in this study to determine the temporal and spatial distribution of *PtRab* (Rab small GTP-binding protein gene) in different lines during ovule development of *P. tabuliformis*. In summary, we further revealed the molecular regulation mechanism of ovule development half-stopping in a female sterile mutated line in *P. tabuliformis* in this study, preliminarily mining the key factors of ovule abortion in gymnosperms at the transcriptional level.

## 2. Results

### 2.1. Ovule Phenotypes of P. tabuliformis

**Figure 1:**
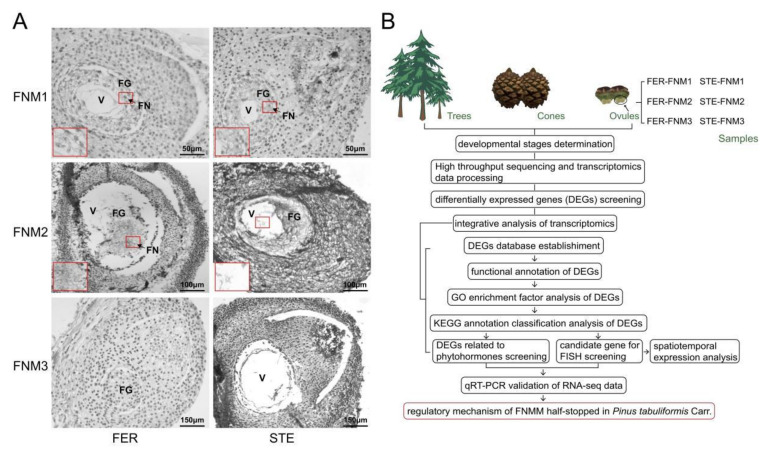
Background description of tissues and workflow. (**A**) Microscopic observations of ovules; FER: female fertile line, STE: female sterile line, FNM: the process of free nuclear mitosis, FG: female gametophyte, FN: free nucleus, V: vacuole. (**B**) Schematic overview of transcriptome data generation and analyses workflow.

The phenotypes of *P. tabuliformis* ovules were shown in Figure 1A. In the early stage of FNMM, the anatomical structures of female sterile line (STE) and female fertile line (FER) ovules were similar. The center of FG was a large vacuole surrounded by several dozen free nuclei. In the middle stage, the free nuclei were still increased in FER; however, this trend was arrested in STE. Up to the late stage, the number of free nuclei was sharply increased and filled the center in FER ovules, while the free nuclei in STE ovules began to disappear and their centers were empty.

### 2.2. Sequencing, Read Assembly and Global Data Analysis

To explore the process of FNM and reveal the reasons of the development of FNMM half-stopped in the female sterile lines of *P. tabuliformis*, we selected three key time points: the early, middle, and late periods of the FNM process. FER ovule samples at the collection periods described above were marked as FER-FNM1, FER-FNM2, and FER-FNM3, respectively, while STE ovule samples were marked as STE-FNM1, STE-FNM2, and STE-FNM3, respectively, and each sample from the above periods was in triplicate (Supplementary Figure S1). A total of 18 mRNA libraries were paired-end (2 × 150 bp) sequenced using the HiSeqTM 4000 platform, resulting in 142.7 gigabase clean reads. The Q30 percentage of each sample was between 97.32% to 97.51%, and the length of N50 was 1767 bp. Given the assembled sequences, a total of 63,449 unigenes were generated from 18 libraries.

**Figure 2:**
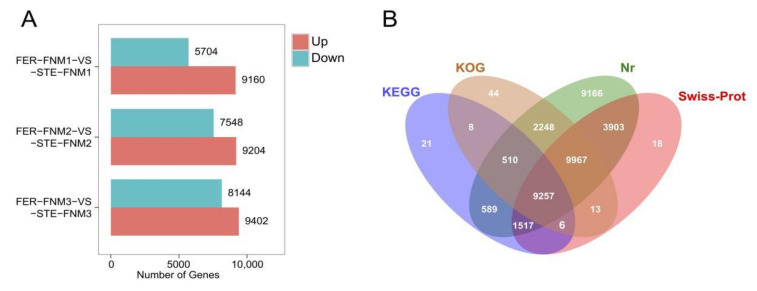
RNA sequencing data from ovules in *P. tabuliformis*. (**A**) Differentially expressed genes between FER and STE in FNM1, FNM2, and FNM3. (**B**) Venn diagram of unigene annotation in *P. tabuliformis* ovules.

The reads mapped to de novo assembly were used for further research, and the mapping ratios of each sample were all beyond 84% (Supplementary Table S1). DEGs (differentially expressed genes) were screened with the FDR value <0.01 and fold change >2. From Figure 2A, we can find that there were more DEGs up-regulated than down-regulated in FER during the whole FNM process. In the FNM1 period, compared with STE, there were 9160 DEGs up-regulated in FER and 5704 down-regulated; in the FNM2 period, 9204 DEGs up-regulated and 7548 down-regulated in FER; and in the FNM3 period, there were 9402 DEGs up-regulated and 8144 down-regulated in FER.

For annotation of the generated unigenes, alignment searches were conducted against public bioinformatics databases. As shown in Figure 2B, among these unigenes, 37,270 unigenes had hits in public databases, including the NCBI non-redundant (NR) database, the Swiss-Prot protein database, Gene Ontology (GO), and the Kyoto Encyclopedia of Genes and Genomes (KEGG). The results indicated that 37,157 unigenes had strong similarity to proteins in the NR database. Similarly, up to 24,684 unigenes had Swiss-Prot annotation, 22,053 unigenes had KOG annotation, and 11,911 unigenes were matched in the KEGG database.

### 2.3. Gene Annotation and Functional Classification

**Figure 3:**
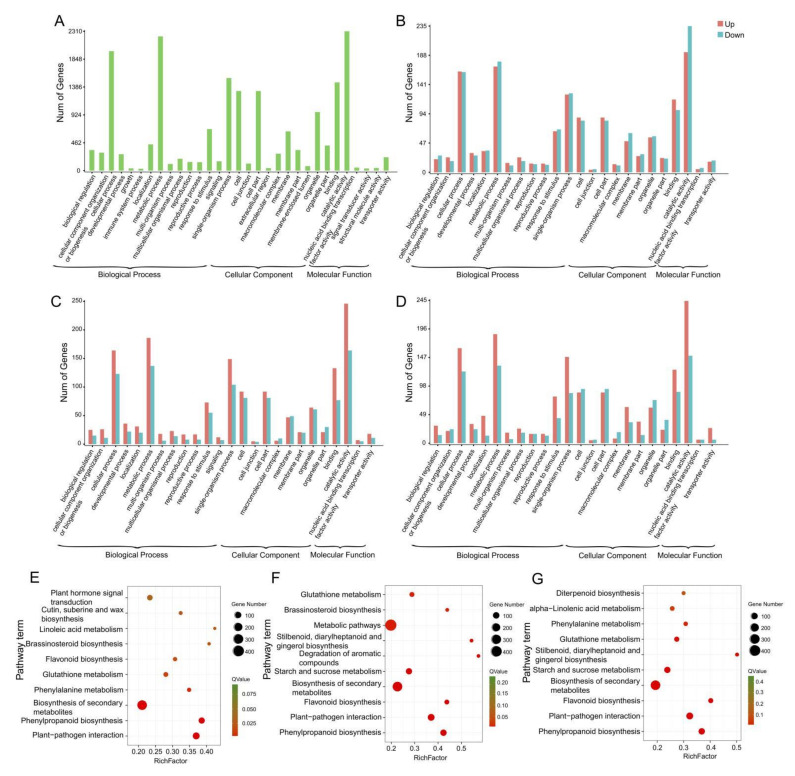
GO and KEGG annotation of unigenes of different free nuclear mitosis stages in ovules of *P. tabuliformis*. (**A**) GO annotation of unigenes of the ovule of *P. tabuliformis*. (**B**–**D**) GO annotation of differentially expressed genes between FER and STE in the stage of FNM1, FNM2, and FNM3 of *P. tabuliformis*. (**E**–**G**) KEGG pathway enrichment of differentially expressed genes in the stage of FNM1, FNM2, and FNM3 of *P. tabuliformis*.

To investigate the expression differences of unigenes between FER and STE, we used reads per kb per million reads (RPKM) to evaluate the expression level of DEGs during the FNM process in two lines, as well as to further reveal the function distribution of unigenes, which were classified by Gene Ontology (GO) annotation (Figure 3A). Annotated unigenes were divided into three categories, “biological process”, “cellular component”, and “molecular function”. In the biological process category, annotated unigenes were significantly enriched in “metabolic processes” (2225), “cellular processes” (1980), and “single-organism processes” (1535). Furthermore, “cell” (1320) and “cell part” (1320) were more obviously enriched in the cellular component categories; in the molecular function category, the unigenes were mainly enriched in “catalytic activity” (2310) and “binding” (1462). To characterize the function distribution of unigenes, we have analyzed GO terms in each ovule development stage in FER and STE (Figure 3B–D, Tables S2–S4). In each period of the FNM process, DEGs between two lines were all mainly enriched in the “cellular process”, “metabolic process”, and “response to stimulus” terms in the biological process category; in the cellular component category, “cell”, “cell part”, and “membrane” terms were dominant; “catalytic activity” and “binding” were still enriched with the most DEGs in the molecular function category.

KEGG was also used to investigate the pathway and network of the molecular regulation in each sample. From Figure 3E–G we found that from the FNM1 to the FNM3 period, DEGs were highly enriched in “phenylpropanoid biosynthesis”, “plant-pathogen interaction”, “biosynthesis of secondary metabolites”, and “flavonoid biosynthesis”. Furthermore, many DEGs were enriched in the “glutathione metabolism” and “brassinosteroid biosynthesis” pathways in the FNM1 and FNM2 periods and were also extremely enriched in “starch and sucrose metabolism” and “stilbenoid, diarylheptanoid and gingerol biosynthesis” in the middle and late periods of FNM.

### 2.4. DEGs Related to Phytohormones in Comparisons between Fertile and Sterile Ovules during FNMM

**Figure 4:**
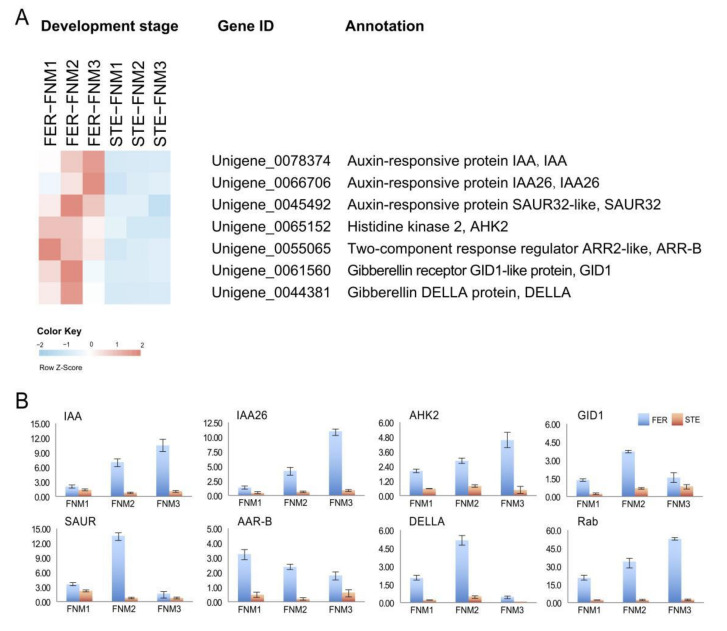
Analysis of expression levels of the differentially expressed genes. Expression patterns of differentially expressed genes related to the plant hormone. Data for the gene expression level were normalized to Z-score (**A**). Candidate DEGs expression levels revealed by qRT-PCR (**B**). Auxin-responsive protein IAA (*IAA*); auxin-responsive protein IAA26 (*IAA26*); auxin-responsive protein SAUR32-like (*SAUR32*); histidine kinase 2 (*AHK2*); two-component response regulator ARR2-like (*ARR-B*); gibberellin receptor GID1-like protein (*GID1*); gibberellin DELLA protein (*DELLA*); Rab-type small GTP-binding protein (*Rab*).

According to the sequencing data and the analysis results of the pathways, we found that many genes participating in plant hormone transport were significantly differently expressed in FER and STE ovules. As shown in Figure 4A, many DEGs related to hormone transport pathways were up-regulated in FER ovules in the FNM process. Specifically, the transcript levels of auxin-related genes (*IAA*, Unigene_0078374, Unigene_0066706; *SAUR*, Unigene_0045492), cytokinin-related genes (*AHK2*, Unigene_0065152; *ARR-B*, Unigene_0071228), and gibberellin-related genes (*GID1*, Unigene_0061560; *DELLA*, Unigene_0044381) were markedly higher in FER than STE.

### 2.5. Verification of the Gene Expression Profile by qRT-PCR

To confirm the transcriptomic analysis results, the DEGs listed above were selected for quantitative real-time PCR (qRT-PCR) validation using the same type of samples compared with formerly used samples in high-throughput sequencing analysis. The primers of these DEGs are listed in Supplementary Table S5. The expression profiles of the candidate DEGs revealed by qRT-PCR data were consistent with those derived from sequencing (Figure 4B). We found that DEGs related to hormones were highly expressed in FER, and the expression levels of *SAUR* and *DELLA* were significantly high in the FNM2 stage. In addition, *Rab* was also up-regulated and expressed more highly in FER. The expression profiles of the qRT-PCR data were consistent with those derived from sequencing.

### 2.6. Expression Patterns of DEGs between STE and FER during the FNM Process

**Figure 5:**
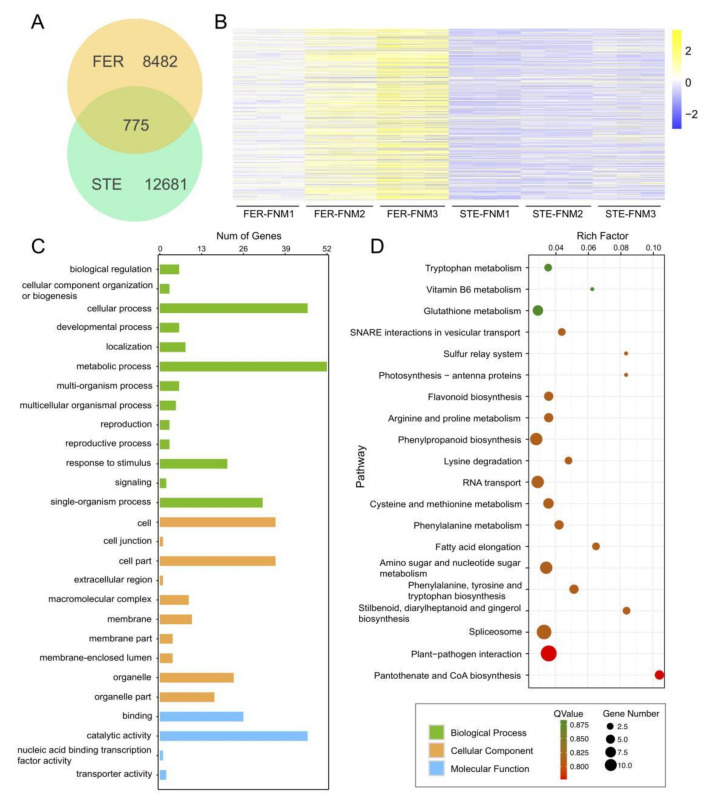
DEGs with opposite expression patterns during the FNM process in STE and FER; DEGs up-regulated in FER and down-regulated in STE, Venn diagram (**A**), and heat map (**B**). GO classification of DEGs with opposite expression levels (**C**). KEGG pathway enrichment of DEGs with opposite expression levels (**D**).

Based on the gene expression levels during the ovule development process in FER and STE, we used Short Time-series Expression Miner (STEM) software to cluster DEGs into eight profiles. These model profiles have been chosen to summarize the expression patterns of the genes. Furthermore, four patterns of genes showed very significant p-values among the eight patterns, marked as colored boxes (Supplementary Figure S2), which contained two up-regulated patterns (profile 6 and 7) and two down-regulated patterns (profile 0 and 1). These DEGs showed contrary expression trends between FER and STE during the FNM process, which might play an indispensable role in the ovule development of *P. tabuliformis*. There were total 775 DEGs up-regulated in FER but down-regulated in STE (Figure 5A,B), and we subjected these DEGs to GO term analysis, which also classified them into three main categories of “cellular component”, “molecular function”, and “biological process” (Figure 5C). Moreover, these DEGs were subjected to KEGG pathway enrichment analysis (Figure 5D), and “pantothenate and CoA biosynthesis”, “plant–pathogen interaction” and “spliceosome” were the top three pathways in which the DEGs were highly enriched.

### 2.7. Spatiotemporal Expression of PtRab in the Ovules

**Figure 6:**
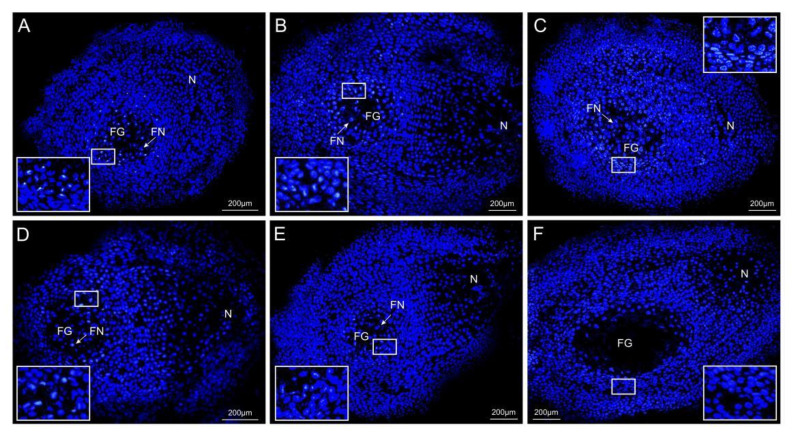
Fluorescence in situ hybridization of *PtRab* in *P. tabuliformis* ovules. (**A**–**C**) The spatiotemporal expression of *PtRab* in ovules from FNM1 to FNM3 period in fertile line. (**D**–**F**) The spatiotemporal expression of *PtRab* in ovules from FNM1 to FNM3 period in sterile line. FG: female gametophyte, FN: free nucleus, I: integument.

Based the expression trend analysis and the result of the qRT-PCR, we found that *PtRab* (Unigene_0044338) was highly expressed in the FER ovules and weakly expressed in STE (Figure 4B). The Rab family is the biggest subfamily of small GTP-binding proteins and participates in a variety of life activities, including protein synthesis, vesicle transport, and cytoskeleton assembly in plant cells. A digoxigenin-labeled DNA probe of *PtRab* was prepared for in situ hybridization of the frozen sections to display the temporal and spatial differences between two lines during the FNM process. As shown in Figure 6, in the FER ovules, *PtRab* was mainly expressed in the female gametophyte and kept being up-regulated as the number of free nuclei increased. However, the expression level of *PtRab* was significantly lower in the STE ovules and kept being down-regulated during ovule development up until the FNM3 stage, where the fluorescence signal was nearly undetectable. A fluorescence signal of *PtRab* was also detected in the surrounding tissue in two lines, but the signal was too scattered, which cannot directly reflect the expression trend in the ovules.

### 2.8. Development and Characterization of SSR Markers

Transcriptome sequencing provided a simple and effective method to develop a large number of unigene-based SSR markers, which was essential for mining a large number of functional SSR markers [18]. To further investigate the differences in regulatory mechanisms between FER and STE ovules, microsatellites were identified in this study. We used a microsatellite (MISA, http://pgrc.ipk-gatersleben.de/misa/, accessed on 23 March 2021) to analyze the transcriptome data, and a total of 3756 potential simple sequence repeat (SSR) markers were identified in 3288 unigenes. 374 unigenes contained more than one SSR; and the frequency of SSRs was 3.83%. As shown in Table 1, potential microsatellites were discovered and defined as di- to hexa-nucleotide motifs. The dinucleotide and trinucleotide repeats ranked first (49.95% and 30.91%, respectively), while the sum of the other type of nucleotide repeats accounted for less than 20%. The most represented repeat units of potential SSRs were 4–12, up to 3724 (99.15%), and 6 repeats and 5 repeats were the most significant (29.47% and 24.2%, respectively). Based on Supplementary Figure S3, we analyzed the SSR motifs in *P. tabuliformis*. The AT/TA di-nucleotide repeat was the most abundant motif (24.8%), followed by AG/CT (16.6%), AC/GT (8.6%), AAG/CTT (6.6%), and AAG/CTT (6.4%). These five types of nucleotide repeats mentioned above represented about 60%.

## 3. Discussion

The development of FG was the key process of plant gametophyte and sporophyte generation alternation, which also affected the development of seeds. So far, most studies on FG development were focused on angiosperms, such as *Arabidopsis thaliana* [19], although there were a few studies on female sterility in woody plants, such as *Ulmus pumila* Mill. and *Xanthoceras sorbifolia* Bunge [20,21]. Nevertheless, due to the fact that the natural female sterile mutants of gymnosperms were scarce and the volume of the ovules was too small, the molecular mechanisms of ovule development in gymnosperms were still unclear. In this study, we used high-throughput sequencing to analyze the transcripts in different periods of FER and STE ovules in *P. tabuliformis*. A total of 18 cDNA libraries were created (Supplementary Figure S1), and we obtained 142.7 G clean data and 98,038 unigenes in total. Analysis results of GO and KEGG indicated that the GO terms’ and KEGG pathways’ DEG enrichment were highly similar in varying lines, demonstrating that the transcriptome at the pathway level was generally conserved in the ovules of *P. tabuliformis* (Figure 3).

SSR markers have been used to determining protein function, genetic development, and gene expression regulation [21]. For example, SSR markers have been used to identify the position of the gene causing female sterility in wheat and soybean [21,22]. As shown in Table 1, we obtained 3756 SSR markers from 3288 unigenes, of which the frequency was 3.83%. The frequency of SSRs was diverse in different species, such as *Codonopsis pilosula* (12.2%) [23], *Populus* L. (15.3%) [24], and *Robinia pseudoacacia* L. (13.79%) [25]. This rate being lower in *P. tabuliformis* might be because of the lack of a reference genome, leading to a lack of information; another reason is that the gymnosperms were relatively primitive, with genes more stable than other species in the long-term evolution process, and not prone to mutation. The frequency of SSRs was also low in some other gymnosperm species, such as *Taxaceae* (2.24%) [26] and *Ginkgo biloba* L. (5.09%) [27]. Similarly to the other plants, excluding the mono-nucleotide repeats, di-nucleotide repeats were the most abundant type, followed by tri-nucleotide repeats [28].

As signal molecules in plants, hormones regulate a variety of biological processes. In this investigation, the plant hormone signal transduction pathway enriched many DEGs, and the exploration of the expression patterns of DEGs related to plant hormones was helpful to further reveal the internal mechanism of the cessation of FNMM in FG. Auxin is a mobile signaling molecule regulating various processes in plant development which also influences the development of ovules [29,30]. Previous studies have shown that a low concentration of auxin may lead to a loss of embryo sac in *Arabidopsis* [31]. *GH3*, *SAUR*, and *AUX/IAA* are the auxin primary response genes [32]. Genes related to auxin response showed obvious differential expression in FER and STE ovules at different stages of FNMM, suggesting that correct expression of auxin-response-related genes is crucial to the development of FG. In our investigation, with FNMM development in FER, *AUX/IAA* and *SAUR* were significantly up-regulated; on the contrary, they were all down-regulated in the STE ovules. *SAUR* was especially highly expressed in the FNM2 period, and the expression levels of *AUX/IAA* were significantly higher in the FNM2 and FNM3 periods. It is suggested that auxin accumulation in the STE ovules may be insufficient, which hindered the development of FG. Moreover, DEGs may further affect the distribution of auxin and the development of ovules (Figure 4).

**Figure 7:**
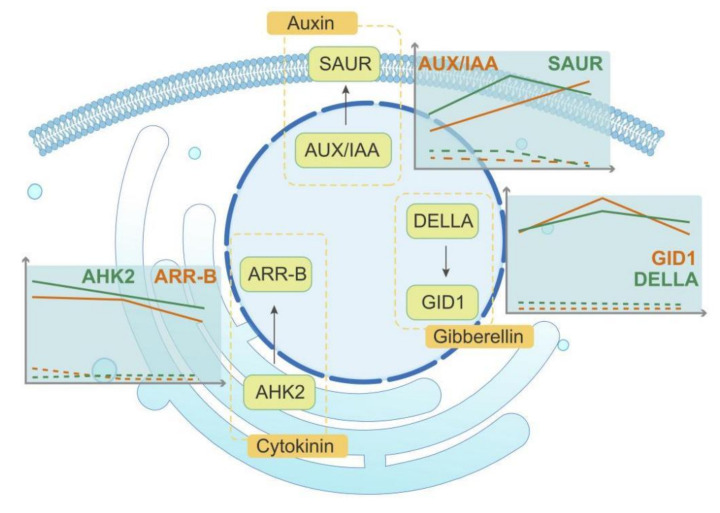
Speculative network model revealing the ovule abortion in the female sterile line of *Pinus tabuliformis* Carr.

Cytokinin is involved in cell division and differentiation, and a decrease in cytokinin concentration in plants might lead to female sterility and a decrease in the number of ovules [33,34]. AHK2 is the receptor protein of cytokinin which undergoes autophosphorylation of histidine after sensing cytokinin [35,36]. Phosphorylated histidine transfer protein has been shown to enter the nucleus, transfer the phosphate group to type-A or type-B response regulators, and then perform a series of regulation on the plants [37,38]. In the present study, many genes associated with the cytokinin pathway showed different expression patterns during ovule development in FER compared with STE. In the FER ovules, *AHK2* and *ARR-B* were up-regulated during the whole FNM process and significantly highly expressed in the FNM1 and FNM2 stages, but in the STE ovules, they were all weakly expressed. This indicated that until the FNM2 period, the free nuclei in the FER ovules maintained vigorous division, whereas the concentration of cytokinin might have been insufficient and affected the nuclear division in the STE ovules (Figure 4). Gibberellin is an important plant hormone which plays an important role in the whole life process of plants and participates in the regulation of plant reproductive growth [39,40]. *GID1* is the receptor protein of gibberellin, and DELLA is another important element protein of gibberellin signal transduction pathway which is also the negative regulator of gibberellin [41,42]. DELLA mainly exists in the nucleus and participated in the regulation of plant growth and reproduction [43,44]. In our investigation, with FNMM development, *GID1* and *DELLA* were significantly up-regulated and highly expressed in the FNM2 period in FER; on the contrary, they were all down-regulated in the STE ovules. It is suggested that the gibberellin-related pathway may have been blocked, which affected the normal development of ovules and induced ovule abortion (Figure 7).

In line with the analysis results of the transcriptome data and expression trends of DEGs, *PtRab* showed obviously different expression between FER and STE. *Rab* plays a pivotal role in protein synthesis, substance transport, and cell division [45]. Previous research showed that *Rab* was abundantly expressed in tissues with cell division, and elongation was vigorous in rice [46]; in *Pinus pinaster* Ait., *Rab* was high expressed in the early stage of embryo development, and the expression level increased when the seed germinated [47]; Gutkowska found that the pollen tube development of *Rab* was abnormal and induced male sterility in *Arabidopsis* [48]. These studies indicated that the expression level of *Rab* perhaps plays a role in the reproductive growth of plants. In this study, we analyzed the temporal and spatial differences of *PtRab* between two lines during the FNM process. We found that in the FER ovules, the expression level of *PtRab* was markedly increased and dominantly expressed in FG; on the contrary, the development of FNMM was disrupted in the FNM2 period in STE, leading to the number of free nuclei decreasing, and *PtRab* was down-regulated without a fluorescence signal detected (Figure 5). Thus, we suspected that *PtRab* might be expressed as FG develops and that a low expression level of *PtRab* might be one the of the causes of female sterility in *P. tabuliformis*.

## 4. Materials and Methods

### 4.1. Plant Materials

In line with the previous analyses from our research group, the FER ovule and STE ovule were genetically closely related by the DNA marker technique [49]. The ovules were selected from the *P. tabuliformis* Seed Garden, Xingcheng, Liaoning Province, China. We selected three time points to collect the samples: the early, middle, and late periods of the FNM process (in early February, March, and April, respectively), and the same size cones were selected and collected from the middle of the crown. The ovules were removed from the scales under a dissecting microscope, and ovules from five cones were collected as one sample. The samples were placed into liquid nitrogen overnight and then stored at −80 °C. Paraffin sections were made according to Zhang et al. [50], and the developmental stages of ovules were observed under a microscope (Leica, Weztlar, Germany).

### 4.2. RNA Sample Preparation and RNA-Seq

We used Plant RNA Assistant Kit (Kebaiao, Beijing, China) to extract the total RNA from ground samples, according to the manufacturer’s instructions, and quantified the integrity of the RNA by agarose gel electrophoresis and Agilent 2100 Bioanalyzer. The purity and concentration of the RNA were determined by Nanodrop 2000 (Thermo Specific, USA).

The RNA-Seq samples were used to construct 18 libraries (FER-FNM1, FER-FNM2, FER-FNM3, STE-FNM1, STE-FNM2, and STE-FNM3; each sample had three replicates). After total RNA was extracted, mRNA was enriched by Oligo (dT) beads and then fragmented into short fragments using fragmentation buffer and reverse transcripted into cDNA with random primers. Second-strand cDNA was synthesized by DNA polymerase I, RNase H, dNTP, and buffer. Then, the cDNA fragments were purified with QiaQuick PCR extraction kit (QIAGEN, Valencia, CA, USA), were end-repaired, had poly (A) added, and were ligated to Illumina sequencing adapters. The ligation products were size-selected by agarose gel electrophoresis, PCR-amplified, and sequenced using Illumina HiSeqTM 4000 (Illumina, San Diego, CA, USA).

### 4.3. De Novo Assembly

The raw reads were cleaned by removing adapter sequences, low-quality sequences (reads with ambiguous bases “N”), and reads with more than 10% Q < 20 bases. De novo assembly of the clean reads was performed using Trinity [51] program to build the reference-free full-length transcription. In the present study, K-mer value 25 was chosen in Trinity, with group-pairs-distance =500 and other default parameters. Next, Trinity connected the contigs between each pair of contigs, using “N” to represent unknown bases, thus generating scaffolds. Paired-end reads were used again for scaffold gap filling to obtain sequences with the fewest Ns and those that could not be extended at either end. Such sequences were defined as unigenes. Finally, the potential transcript sequences were clustered using the TGI Clustering tool to obtain uni-transcripts [52].

### 4.4. Functional Annotation

We BLAST the genes based on four databases to annotate the unigenes: the Nr database (http://www.ncbi.nlm.nih.gov, accessed on 3 October 2020), the KEGG database (http://www.genome.jp/kegg, accessed on 3 October 2020), the Swiss-Prot protein database (http://www.expasy.ch/sprot, accessed on 6 October 2020), and the COG/KOG database (http://www.ncbi.nlm.nih.gov/COG, accessed on 6 October 2020), with BLASTx program (http://www.ncbi.nlm.nih.gov/BLAST/, accessed on 6 October 2020) to evaluate sequence similarity with genes of other species at an *e*-value threshold of 1×10^−5^. GO annotation was analyzed by Blast2GO software [53] with Nr annotation results, and WEGO software was used to perform the functional classification of unigenes [54]. The KEGG database was used to explore the genes involved in the biological pathways, and the pathway was defined as significantly enriched with *p*-value <0.05. All raw data of high-throughput sequencing have been deposited to the National Genomics Data Center (https://bigd.big.ac.cn, accessed on 25 January 2022) with the dataset accession number PRJCA004609.

### 4.5. Analysis of Differentially Expressed Genes (DEGs)

We estimate the gene expression level by the reads per kilobase of exon model per million mapped reads (RPKM) [55,56]. To identify DEGs across samples, the edgeR package (http://www.r-project.org/, accessed on 27 October 2020) was used. We identified genes with a fold change ≥2 and a false discovery rate (FDR) <0.01 in a comparison as significant DEGs.

### 4.6. Simple Sequence Repeat (SSR) Marker Prediction

We used MISA (http://pgrc.ipk-gatersleben.de/misa/, accessed on 30 October 2020) to discover the SSRs in the whole transcriptome. The minimum number of repeat units was as follows: six for di-nucleotides, five for tri-nucleotides, four for tetra-, penta-, and hexa-nucleotides. The shortest distance between two SSRs was 100 bp, or else they would be seen as one SSR.

### 4.7. Fluorescence In Situ Hybridization

#### 4.7.1. Preparation and Labeling of DNA Probes

Primer for PtRab was 5′-primer CTTCTAAAGGCAACATTC, 3′-primer TCTATCCCTACACTGGTT. The cDNA of P. tabuliformis ovule was used as template for PCR and purified by Cycle Pure Kit (Biomiga, San Diego, CA, USA). DNA probe was marked by PCR DIG Probe Synthesis Kit.

#### 4.7.2. Preparation of Frozen Sections

Successive sections of 10 μm were made at −20 °C with Leica CM1950 freezing microtome (Leica, Weztlar, Germany), cutterbed angle is 8°. The sections were picked up with an anatomical needle and adhered to the slide; the glass slide was specially used for FISH (Superfrost^®^ Plus Slide, Hatfield, PA, USA). Dried at room temperature for 1–2 h.

#### 4.7.3. Pretreatment of Frozen Sections before the Fluorescence In Situ Hybridization

The dried frozen sections were put into the hybridization tank, hydration of 10 mM sodium citrate buffer 5 min, and then transferred to equal volume 2 × SSC solution for 5 min; finally, sections were transferred to 50% formamide prepared by 2 × SSC for 1 h.

#### 4.7.4. Fluorescence In Situ Hybridization

Hydrated frozen sections with 10 mM sodium citrate buffer for 5 min, immersed in 2 × SSC solution for 5 min, stood in 50% formamide for 1 h, and then put into a slice box containing 100% ethanol, avoiding dry slices. Attached the hybridization chamber to the slide and protected from light strictly with the following steps: add 20 µL of pre-hybridization solution without probe and pre-hybridize at 37 °C for 2 h; remove the probe and heat it at 85 °C for 5 min to denature the probe; add probe to the small hole left in the hybridization chamber and hybridize at 37 °C 12—24 h.

#### 4.7.5. Elution and Detection after Hybridization

Removed the hybridization chamber, rinsed in 2 × SSC solution at 37 °C (5 min/time, 3 times), 0.1 × SSC rinsing solution, (5 min/time, 2 times), 2 × SSC solution isotonic for 2 min. After natural drying, 0.05 μg/mL DAPI was added dropwise to cover the tissue section and counterstained for 30 min. Wiped the slide and diluted the anti-digoxin antibody at 1:1000, and then, added Antifade Mounting Medium (Absin, Shanghai, China) and mounted. Finally, observed with a fluorescence microscope by ultraviolet fluorescence.

### 4.8. Real-Time Quantitative PCR Analysis

The Plant RNA Assistant Kit was used to isolate the total RNA in this research (Kebaiao, Beijing, China). Reverse transcription into cDNA was performed with SuperReal PreMix Plus (TIANGEN Biotech, Beijing, China), performed following the protocols included with the kits. Primers were designed using Primer Premier 6. The qRT-PCR was performed using a MiniOpticon Real-Time PCR Detection System (Bio-Rad, Hercules, CA, USA). The PCR condition was 94 °C for 35 s, then 45 cycles of 95 °C for 3 s for denaturation, and 55 °C for 30 s for annealing and extension, repeated three times. Relative expression levels of the target genes were normalized and then calculated using comparative Ct (2^−ΔΔ^ Ct) method.

## 5. Conclusions

In this study, we analyzed transcriptome features based on high-throughput sequencing in different FNM periods of the FER and STE ovules in *P. tabuliformis*. Our findings determined the DEGs associated with plant hormone, suggesting that the synthesis of auxin, cytokinin, and gibberellin might be abnormal in STE ovules. In addition, the spatiotemporal expression of *PtRab* was different between FER and STE, indicating that *PtRab* might play an important role during ovule development. These findings may provide new strategies for improving our understanding of the molecular mechanisms involved in the FNM process and ovule development in gymnosperms (Figure 7).

## Figures and Tables

**Table 1:** Occurrence frequency of different microsatellite repeat motifs of *P. tabuliformis* ovule transcriptome.

Repeat Motif Length	Repeat Number										Total	Percentage
	4	5	6	7	8	9	10	11	12	>12		
Di-nucleotide			795	362	294	193	128	57	15	32	1876	49.95%
Tri-nucleotide		821	212	92	22	8	3	0	3		1161	30.91%
Tetra-nucleotide	349	50	1	1			1				402	10.7%
Penta-nucleotide	132	10	1		1						144	3.83%
Hexa-nucleotide	140	28	2	1	2						173	4.61%
Total	621	909	1011	456	319	201	132	57	18	32	3756	100%
Percentage	16.53%	24.2%	29.47%	12.14%	4.49%	5.51%	3.61%	1.52%	0.48%	0.85%		

## Data Availability

All raw data of high-throughput sequencing have been deposited to the National Genomics Data Center (https://bigd.big.ac.cn, accessed on 25 January 2022) with the dataset accession number CRA004027.

## Supplementary Materials

The following supporting information can be downloaded at: https://www.mdpi.com/article/10.3390/ijms23031915/s1.
