# Supplementary material for: Screening of Differentially Expressed Genes and Localization Analysis of Female Gametophyte at the Free Nuclear Mitosis Stage in Pinus tabuliformis Carr
Source: Int J Mol Sci. 2022 Feb 8;23(3):1915. doi: 10.3390/ijms23031915 (PMC8837038; doi:10.3390/ijms23031915)
Supplement: Supplementary file 1 [file ijms-23-01915-s001.zip › Figure S2.pdf]

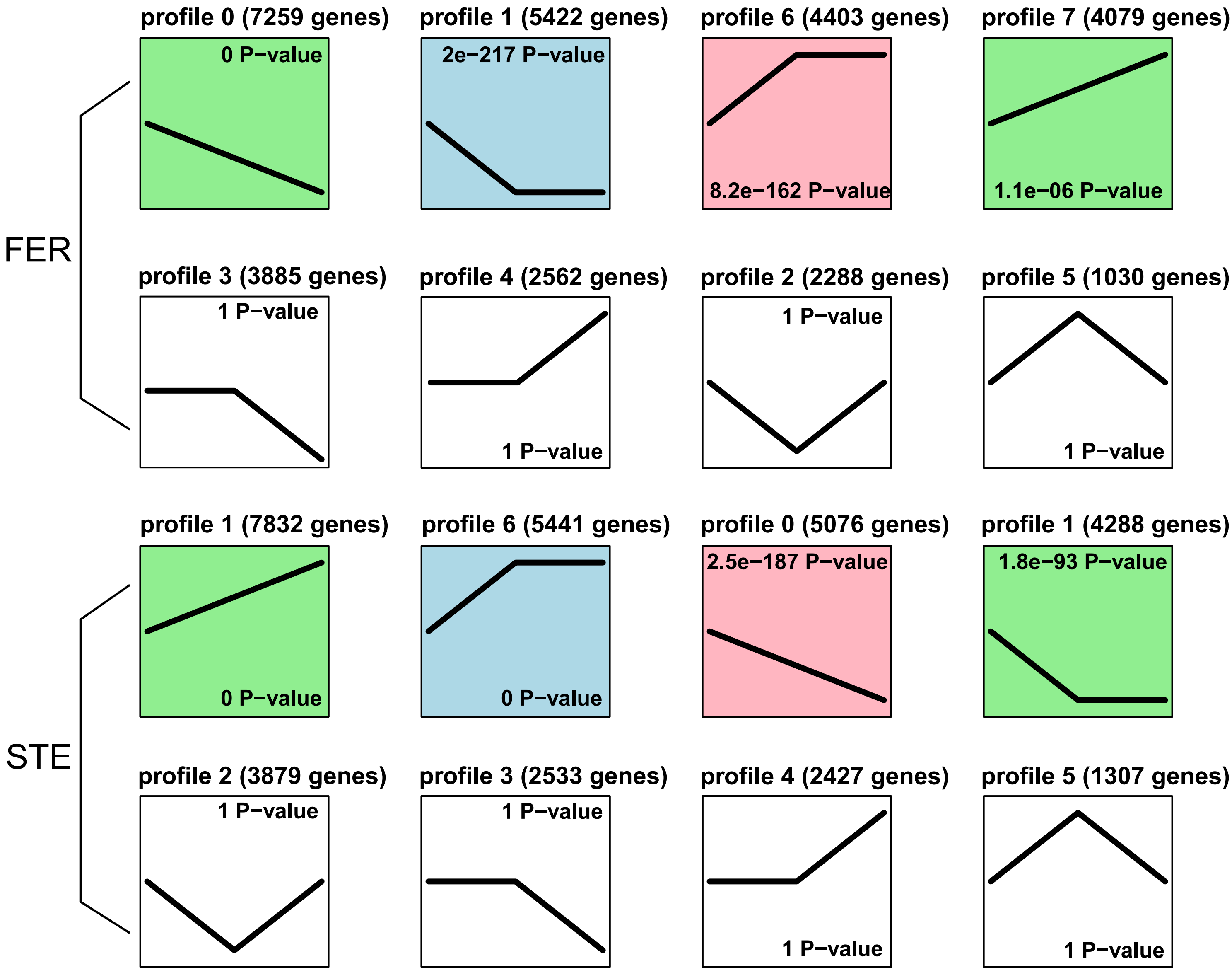
FER
profile 0 (7259 genes)
0 P−value
profile 1 (5422 genes)
2e−217 P−value
profile 6 (4403 genes)
8.2e−162 P−value
profile 7 (4079 genes)
1.1e−06 P−value
profile 3 (3885 genes)
1 P−value
profile 4 (2562 genes)
1 P−value
profile 2 (2288 genes)
1 P−value
profile 5 (1030 genes)
1 P−value
STE
profile 1 (7832 genes)
0 P−value
profile 6 (5441 genes)
0 P−value
profile 0 (5076 genes)
2.5e−187 P−value
profile 1 (4288 genes)
1.8e−93 P−value
profile 2 (3879 genes)
1 P−value
profile 3 (2533 genes)
1 P−value
profile 4 (2427 genes)
1 P−value
profile 5 (1307 genes)
1 P−value
