# Supplementary figures and images for: Screening of Differentially Expressed Genes and Localization Analysis of Female Gametophyte at the Free Nuclear Mitosis Stage in Pinus tabuliformis Carr
Source: Int J Mol Sci. 2022 Feb 8;23(3):1915. doi: 10.3390/ijms23031915 (PMC8837038; doi:10.3390/ijms23031915)

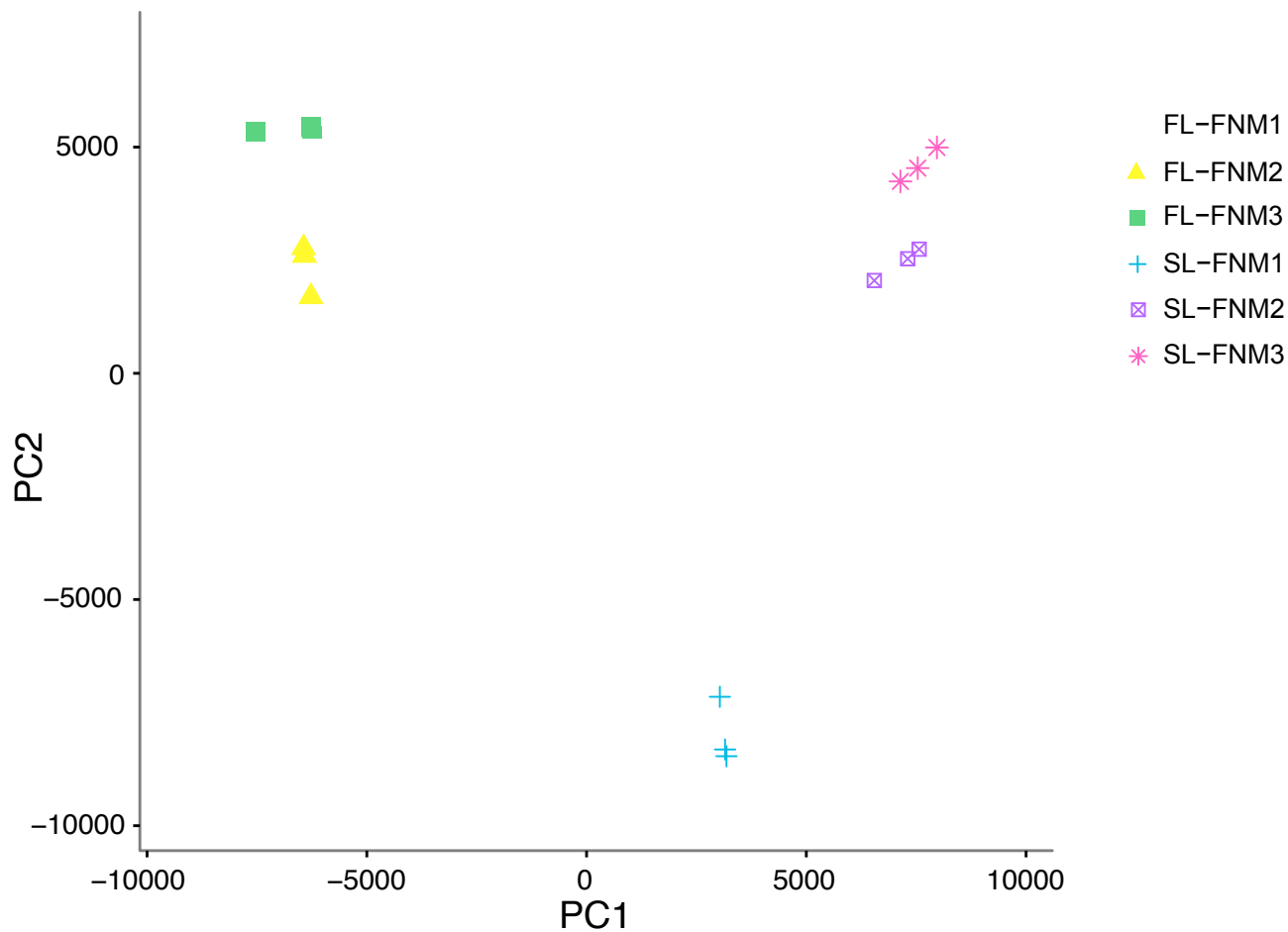

Supplement: Supplementary file 1 [file ijms-23-01915-s001.zip › Figure S1.pdf]

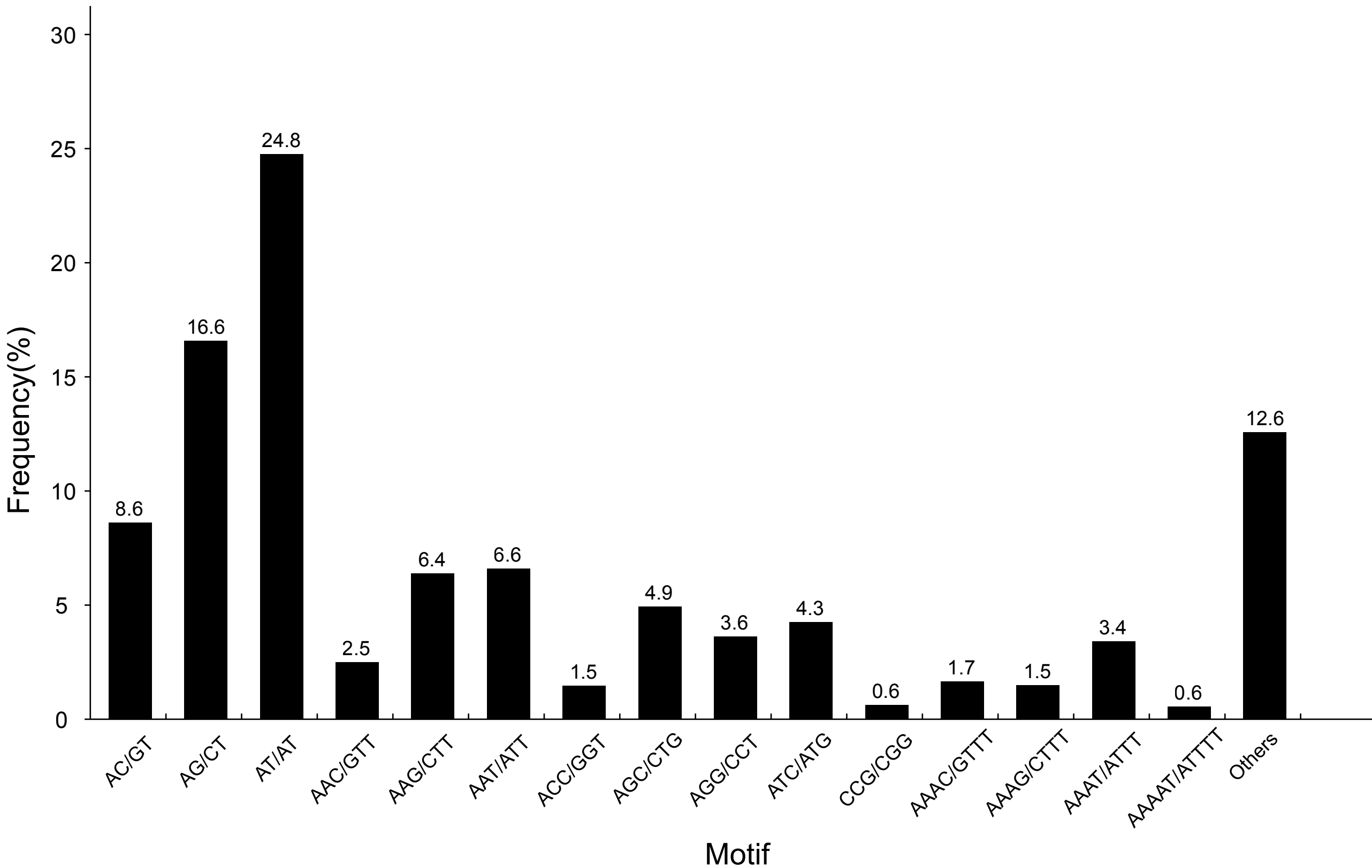

Supplement: Supplementary file 1 [file ijms-23-01915-s001.zip › Figure S3.pdf]
